# Waste Bread as Main Ingredient for Cookie Elaboration

**DOI:** 10.3390/foods10081759

**Published:** 2021-07-29

**Authors:** Priscila Guerra-Oliveira, Mayara Belorio, Manuel Gómez

**Affiliations:** Food Technology Area, College of Agricultural Engineering, University of Valladolid, 34071 Palencia, Spain; priscilaguerra92@gmail.com (P.G.-O.); beloriom@gmail.com (M.B.)

**Keywords:** stale bread, food waste recovery, cookies, circular economy

## Abstract

Food waste is a current global problem. The aim of this work was to investigate the possibility of reintroducing bread discarded by retailers in the preparation of sugar-snap cookies. Bread flours were obtained from stale breads (white and whole wheat) milled with 200, 500 and 1000 μm sieves. Cookies were elaborated using 100% bread flours and combinations of 50% of bread flour and wheat flour. The rheology of the doughs, the dimensions of the cookies, their texture and colour were evaluated. Bread flour doughs presented higher G’ (elastic modulus), G” (viscous modulus) values than the control, especially with increased particle size. Bread flour cookies had a smaller diameter and a harder texture than the control, but in the case of whole bread flours of larger particle sizes, those differences were reduced. Cookies made with bread flour had a darker colour and higher a* values. The 50% mixtures did not present significant differences with respect to the control in terms of dough rheology, hardness, or lightness. Although the spreading factor was reduced, it was more similar to the control than to 100% bread flour cookies. Wasted bread flour can thus be used to replace wheat flour in cookie formulations.

## 1. Introduction

An estimated one third of the food produced in the world is wasted. This waste generates a high expenditure of resources, soil, energy, chemicals and materials needed along the value chain. It also produces a high environmental impact that might be reduced by minimizing food waste or through better food products management practices [1]. In recent years, and especially after COVID19, there is an increased awareness of the importance of sustainability in the food chain to avoid or reduce the frequency of relevant food and health crises in the future [2,3]. Among the food wasted in supermarkets those with the highest environmental footprint are meat and bakery products [4]. It has been shown that the correct management of waste bread is the best measure to overcome the increase of the greenhouse effect when compared to other wasted food items [5]. Notwithstanding the significant concern about bread waste in industry and retailers, studies which support the reintroduction of this waste into the human food chain are very scarce and limited to its use for sourdough production [6] and extruded snacks [7,8].

Bread is a product formulated mainly with flour and water. Due to the fermentative processes and the high temperature bread is submitted to, flour undergoes important modifications during the baking process. The high temperature of baking favours the denaturation of proteins and the gelatinisation of starch [9]. Therefore, flour components lose some of their functional properties, such as the ability to form a gluten network or to gelatinise. In addition, Maillard reactions between proteins and amino acids occur in the crust, modifying the colour, flavour and aroma [10].

Fortunately, breads with lean formulas usually harden before there is any appreciable development of microorganisms, so they are discarded without a high microbial load and can be milled to be reused in the form of flour or semolina.

In the production of sugar-snap cookies the gluten network is not developed, nor does the starch gelatinise, due to the absence of sufficient water in the formulation. In fact, these cookies can be elaborated with flours that have undergone heat treatment [11]. Flours obtained through the milling of stale bread could therefore be used in the production of this type of cookies. In addition to the type of bread, it will be necessary to consider the particle size of the flours, since it influences the rheology of the doughs, the spreading factor, and the texture of the cookies [12].

The aim of this work was to evaluate the use of flours obtained from stale breads in sugar-snap cookies. For this purpose, two different bread types (traditional white and whole wheat) were ground with different sieves (200, 500 and 1000 μm) to obtain flours of three different particle sizes. Flour colour, dough rheology and dimensions, as well as cookie texture and colour were analysed. A focus group discussion has also been included to evaluate the sensory quality of the obtained cookies.

## 2. Materials and Methods

### 2.1. Materials

The following ingredients have been used in the preparation of the different cookies: wheat flour (10.03 g moisture/100 g flour; 8.98 g protein/100 g flour) provided by Harinera Castellana S.L. (Medina Del Campo, Valladolid, Spain), traditional white bread (wheat; water; salt; yeast; malt.) and whole wheat bread (whole wheat; water; wheat gluten; salt; yeast; vegetable extracts; esters mono- and diacetyl tartaric acid esters of mono- and diglycerides; lactic acid; ascorbic acid). purchased at Carrefour (Palencia, Spain), sucrose (AB Azucarera Ibérica, Valladolid, Spain), Argenta “cream” margarine (Puratos, Girona, Spain), sodium bicarbonate (Manuel Riesgo S.A., Madrid, Spain) and local potable water.

### 2.2. Methods

#### 2.2.1. Bread Flour Preparation

Breads were bought and allowed to dry at room temperature for 24 h prior to milling. Bread flours were obtained using 100% of traditional white bread (TBF) or 100% of whole wheat bread (WBF). Breads were ground with a ZM 200 ultra-centrifugal mill (Retsch, Düsseldorf, Germany) equipped with three different sieves (200, 500 and 1000 μm) to obtain flours of different particle sizes. These particle sizes were evaluated using a Mastersizer 3000 (Malvern Instruments, Malvern, UK) and values of D [3,4], which accounts for the equivalent spherical diameter of the particles, were obtained. The final flour samples were identified as: control wheat flour (111 µm), TBF200 (170 µm), WBF200 (153 µm), TBF500 (276 µm), WBF500 (270 µm), TBF1000 (306 µm), WBF1000 (291 µm).

#### 2.2.2. Cookie Making Procedure

Nine different sugar-snap cookies were elaborated. Control was prepared with wheat flour and six samples with 100% of each bread flour. Two other samples were elaborated with 50% of the flours (wheat flour/TBF1000 and wheat flour/WBF1000). These cookies have been called TBF1000 50% and WBF1000 50%, respectively. The cookie control formulation (CF) and the eight different cookies with bread flour were prepared according to the recipe presented in Table 1. The amount of water used was adjusted to flour moisture content to 15.0%, and the proportion of the flour.

To prepare the cookie, margarine was heated in a microwave for 1 min (1000 watts) and creamed with sugar at speed 4 for 180 s in a Kitchen Aid 5KPM50 mixer (Kitchen Aid, Benton Harbor, MI, USA) with a flat beater, scraping down every 60 s. Then, water was added, and the batter was mixed at speed 4 for 120 s. The finals ingredients added were flour and sodium bicarbonate. Mixing was performed at speed 2 for 120 s, scraping down very 30 s. After mixing, the dough was allowed to stand for 30 min before lamination in a Salva L-500-J sheeter (Salva, Lezo, Spain). Samples were cut (6 mm thick; 40 mm diameter) and taken to the oven (Salva) at 185 °C for 14 min. The cookies were cooled for 60 min before stored in properly closed plastic bags at a 20 °C food chamber. All cookies were prepared in duplicate.

#### 2.2.3. Cookie Dough Rheology

The rheological behaviour of the dough was measured with a rheometer Thermo Scientific HaakeRheoStress 1 (Thermo Fisher Scientific, Schwerte, Germany) and a Phoenix II P1-C25P water bath (Thermo Fisher Scientific, Waltham, MA, USA) that controlled the analysis temperature (set at 25 °C). The rheometer was equipped with a titanium parallel plate geometry sensor PP60 Ti (60 mm diameter and 3 mm space). For the rheology test, doughs of 3 mm thick and 60 mm diameter were used. Panreac vaseline oil was applied (Panreac Química S.A., Castellar del Valles, Spain) on exposed surfaces to prevent samples from drying out during the test. Samples were left at initial position for 300 s prior to the measurements. The tests were then started by a strain sweep test (0.1–100 Pa) at a constant frequency (1 Hz) to identify the linear viscoelastic region of the sample. The strain value was then employed to carry out a frequency sweep test (10–0.1 Hz), which led to the results of elastic modulus (G′ (Pa)), viscous modulus (G″ (Pa)), complex modulus (G* (Pa)) and loss factor (tan δ). Two repetitions were performed and each of them was analysed in duplicate.

#### 2.2.4. Cookie Characteristics

Cookie characteristics were determined 24 h after their elaboration. Width and thicknesses were measured with a calliper. The diameter of each cookie was measured twice perpendicularly, to calculate the average diameter. The spreading factor was calculated by dividing the average width by the thickness. Colour of flour and cookies were measured with a PCE-CSM 2 colourimeter (PCE Instruments, Hochsauerland, Germany) using the D65 illuminant with a 2° Standard Observer. Flour was placed into a cylindrical plastic recipient with 30 mm diameter and 15 mm height so that the samples could be considered as an infinite solid. Five samples of each flour were analyzed at the central point. Six cookies of each formulation were measured at the centre of the surface in two different batches (6 × 1 × 2). The results were expressed in the CIE L* a* b* colour space [13].

Texture was analysed using a TA-XT2 texture analyser (Stable Microsystems, Surrey, UK) and a sounding probe HDP/3PB with a test speed of 2.0 mm/s. The hardness of the cookie was measured by the maximum force required to break the sample. The analysis of dimensions, colour and texture were carried out on six cookies of each elaboration.

#### 2.2.5. Consumer Test

A consumer test was performed in a hedonic sensory evaluation with a sample of 97 volunteer cookie consumers aged from 16 to 60 years old. This evaluation was carried out in individual booths at the Sensory Science Laboratory of the Agricultural Engineering College at the University of Valladolid, Palencia, Spain. These analyses were carried out according to the protocol previously approved by the Committee of Tests and Research from the Hospital Rio Carrión (Palencia, Spain). Cookies were evaluated using a 9-point hedonic scale, ranging from score 1 for extreme dislike to score 9 for extreme like. Appearance, texture, taste, odour and the overall acceptability of five cookies were evaluated (control; TBF1000 50%; WBF1000 50%; TBF1000 and WBF1000). Samples were presented on white plastic dishes coded with four-digit random numbers and served in random order. Water was available for drinking between samples. Cookies were elaborated 24 h before and stored in plastic bags at 20 °C.

#### 2.2.6. Statistical Analysis

To perform the statistical analysis, the Statgraphics Centurion XVI software (StatPoint Technologies Inc, Warrenton, VA, USA) was used. The data for the different variables were processed through an analysis of variance (simple ANOVA), using the Fisher LSD test to describe significant differences between averages at a significance level of *p* < 0.05. A multivariate analysis was used to determine whether there are correlations between some of the different parameters studied.

## 3. Results

### 3.1. Dough Rheology

As shown in Table 2, cookie doughs prepared with bread flour have higher G’, G”, and G* values and lower tan δ than those made with wheat flour. However, the fact that the doughs made with 50% flour mixtures do not differ significantly from those made with wheat flour is noteworthy. Concerning the type of bread, no significant differences were found between the doughs obtained with TBF and WBF considering the same particle size. Nevertheless, there is a trend towards an increase in the values of G’, G”, and G* and a decrease in tan δ as the particle size of the flours goes up.

In bread making, starch gelatinisation occurs [9,14], when a flour mixture (containing starch) with sufficient hydration is heated [15]. This gelatinisation increases such flour hydration properties [16,17] as water holding capacity (WHC) and water binding capacity (WBC). According to Camire et al. [18], integrity rupture of starch granules leaves numerous hydroxyl groups capable of binding exposed water molecules. It is also known that, in general, higher WHC or WBC values of flours, the higher G’ and G” values in cookie doughs [11,19,20]. This would explain the differences between the rheology of doughs elaborated with wheat flour and those made with bread flour. For a similar reason, doughs elaborated with mixtures of wheat and bread flours is expected to have higher G’ and G” values. Table 2 shows that the average values of G’ and G” do comply with this trend, but there are no significant differences between them and the control. This may be due to the larger deviations detected in measurements with high G’ and G” values, which affect the entire analysis. However, it seems clear that although there may be an increase in rheological values when bread flour is incorporated, there is a point at which this increase peaks, and this is above 50%. This effect could be influenced by the particle size of the flours. As Belorio et al. [12] showed, when increasing the content of larger particles in the flour, cookie doughs become more brittle and G” and G” values increase. This effect would explain the differences between doughs containing bread flour with different particle size. But when the percentage of fine flours is increased, more compact doughs were produced, and they also had higher G’ and G” values. In this case it could compensate the effect of the incorporation of bread flours in doughs made from mixtures of different flours.

### 3.2. Cookie Characteristics

Table 3 shows the dimensions and the texture of the different cookies. In general, as the percentage of bread flour increased, the thickness increased but the diameter of the cookies and the spreading factor decreased. There also seems to be a tendency for the diameter to increase when the particle size becomes progressively greater, although this does not translate into variations in the spreading factor. There were no significant differences between the diameter and the spreading factor from WBF or TBF with the same particle size. However, cookies made with WBF were slightly thicker considering the same particle size, except for the thinner ones, where no significant differences were found.

There is a clear correlation between rheology results and cookie size. Thus, the lower the value of G’, the larger the cookie diameter (r = −0.77) and spread ratio (r = −0.82), and the greater the cookie thickness (r = 0.81). Similarly, the lower the G” value, the greater the cookie diameter (r = −0.84) and spreading factor (r = −0.88), and the greater the cookie thickness (r = 0.81). Also, the highest correlation was observed between the tan δ and the spread ratio (r = 0.93). This correlation is logical since cookie dough expands in the oven according to its rheological properties; the greater the expansion, the lower the resistance of the dough to flow [11,20,21,22,23].

Regarding texture, cookies elaborated with bread flour were harder than those made with wheat flour. However, it should be noted that cookies with 50% mixtures did not differ significantly from those elaborated with wheat flour. In the case of TBF, there were no significant differences between the hardness of cookies elaborated with flours of different particle size. However, in the case of WBF, there was a progressive decrease in cookie hardness as the particle size of the flours increased.

In this study, cookie hardness correlated 99% with both diameter (r = −0.87) and spreading factor (r = −0.80). It seems logical, therefore, that cookies that expanded less and were more compact were the hardest ones. This correlation has been observed previously [12,23]. On the other hand, the decrease in hardness of WBF with the increase in particle size might be caused by bran particles that interrupt the structure of the cookies. Sozer et al. [24] observed that when wheat bran was incorporated into cookies, hardness was lower when bran particle size was larger. Similar results were observed with the addition of apple pomace, a product with a high fibre content akin to wheat bran, which has different particle sizes [23].

In terms of colour (Table 4), there were no major differences or clear trends between the results, although it was evident that a* value presented lower values, that means less red tone in cookies with wheat flour than in the other cookies. Regarding lightness, control presented the highest values, but they only showed significant differences in cookies made with WBF and intermediate particle size TBF.

In general, bread flours showed higher a*, b* values than the control, and lower lightness values. These results might be related to the presence of the crust in the flours that are darker due to the Maillard reactions that occur during baking. Therefore, the colour of the flours (Table 4) did not significantly influence the L* values of the cookies, probably because of Maillard reactions and sugar caramelization, central processes that account for colour characteristics in cookies [25]. These reactions generate colours similar to those found on bread crusts, compensating and masking the differences found in the colour of the flours. This compensation is especially important for L* and b* parameters. The lightness of the flours might influence the results of WBF. In the same way, the colour of the flours seems to influence the a* parameter, as a similar trend can be seen in both flour and WBF cookie colour, although in the case of cookies with higher a*. The influence of flour colour has already been observed in other studies both with maize flours [26] and with the incorporation of other flours [27].

### 3.3. Consumer Test

The sensory acceptability results are shown in Table 5. Samples prepared with 50% bread flour shown an increased acceptability. This effect is observed in all analyzed parameters, except for the aroma, where no statistical difference was found. Some participants recognized a caramel smell. During bread preparation, starch hydrolysis occurs and sugars are generated; these sugars, although partially consumed by the yeast, can remain in the final bread, enhancing the aroma when heated at high temperatures. Nevertheless, cookie samples with 100% bread flour presented the worst acceptability when compared to the control sample. This results where even more pronounced for the 100% whole wheat bread flour sample. The taste of WBF1000 cookies was described as the worst among the others, with comments about bitterness. Maillard reactions can influence the production of bitter compound with off-tastes that can influence negatively the final product acceptability [28]. This perception also corresponded to a darker colour sample, possibly contributing to worst results for appearance and taste. The negative evaluation of the cookies with 100% bread flour was also influenced by the higher hardness values obtained.

## 4. Conclusions

It is possible to produce cookies with good sensory properties by using stale bread flours instead of wheat flour. If the cookies are made from bread flour only (TBF or WBF), the elastic and viscous components of the dough increase, the expansion during baking is reduced, and the hardness of the cookie increases. However, with 50% flour replacements the results obtained are very similar to the control. Furthermore, there are few differences between using a traditional bread flour or wholemeal bread flour in terms of oven expansion, colour, and texture of the cookies, although there may be differences in the sensory and nutritional aspects. It should also be noted that for the cookie making process the product does not need to have an excessively thin milling, and it may even be better if it is not; a 1000 μm sieve should be sufficient.

## Figures and Tables

**Table 1:** Cookie formulations presented in grams with different percentages of bread flour and particle size.

Ingredients	CF	TBF 1000 50%	TBF 1000	TBF 500	TBF 200	WBF 1000 50%	**WBF 1000**	**WBF 500**	**WBF 200**
Wheat Flour	173.2	86.6	-	-	-	86.6	-	-	-
Bread Flour	-	86.6	173.2	173.2	173.2	86.6	173.2	173.2	173.2
Sucrose	124.8	124.8	124.8	124.8	124.8	124.8	124.8	124.8	124.8
Margarine	77.6	77.6	77.6	77.6	77.6	77.6	77.6	77.6	77.6
Sodium Bicarbonate	3.6	3.6	3.6	3.6	3.6	3.6	3.6	3.6	3.6

CF: control formulation; TBF: traditional bread flour; WBF: whole wheat bread flour. “-”: not used in the preparation.

**Table 2:** Rheological parameters of the cookie doughs.

	G’ (×103)	G” (×103)	G* (×103)	tan δ
CF	123.85 ± 0.07 a	40.50 ± 1.18 a	130.40 ± 0.28 a	0.32 ± 0.01 d
TBF1000 50%	140.55 ± 16.89 a	44.02 ± 4.54 a	147.40 ± 17.39 a	0.31 ± 0.01 d
WBF1000 50%	151.95 ± 6.85 a	47.22 ± 1.64 a	159.30 ± 6.08 a	0.31 ± 0.02 d
TBF200	653.85 ± 225.92 bc	101.49 ± 20.66 b	662.95 ± 225.35 bc	0.16 ± 0.04 bc
TBF500	777.50 ± 107.36 c	102.70 ± 5.27 b	784.90 ± 105.24 c	0.13 ± 0.08 ab
TBF1000	1098.50 ± 98.28 d	137.60 ± 20.93 d	1108.50 ± 101.11 d	0.12 ± 0.01 ab
WBF200	506.05 ± 99.20 b	94.34 ± 3.69 b	515.60 ± 99.13 b	0.19 ± 0.03 c
WBF500	840.95 ± 144.32 c	109.75 ± 4.59 bc	848.80 ± 143.68 c	0.13 ± 0.02 ab
WBF1000	1125.00 ± 123.87 d	127.90 ± 6.78 cd	1133.00 ± 122.03 d	0.11 ± 0.04 a

CF: control formulation; TBF: traditional bread flour; WBF: whole wheat bread flour. G’: elastic modulus; G”: viscous modulus; G*: complex modulus; tan δ: quotient (G”/G’). Rheology of the laminated 3 mm thickness and 60 mm diameter dough. The values with the same letter in the same column do not present significant differences (*p* < 0.05).

**Table 3:** Physical properties of cookies.

	Dimensions			Texture	
	Diameter (mm)	Thickness (mm)	Spreading Factor	Hardness (N)	Firmness (N/mm)
Control	58.17 ± 0.08 e	7.08 ± 0.03 ab	8.22 ± 0.01 c	23.45 ± 5.92 abc	48.91 ± 9.26 a
TBF1000 50%	54.54 ± 0.31 d	7.00 ± 0.25 a	7.80 ± 0.23 b	19.30 ± 0.77 a	61.29 ± 3.51 ab
WBF1000 50%	54.35 ± 0.70 d	7.30 ± 0.21 abc	7.46 ± 0.30 b	20.00 ± 0.57 ab	53.08 ± 8.34 a
TBF200	40.52 ± 0.21 a	7.41 ± 0.05 bc	5.48 ± 0.01 a	47.98 ± 0.76 d	103.91 ± 9.44 d
TBF500	42.77 ± 0.01 abc	7.39 ± 0.11 bc	5.79 ± 0.08 a	42.87 ± 0.99 d	98.31 ± 5.73 d
TBF1000	43.34 ± 0.16 bc	7.48 ± 0.03 cd	5.80 ± 0.05 a	41.45 ± 2.18 d	96.18 ± 9.09 cd
WBF200	41.26 ± 0.25 ab	7.54 ± 0.05 cde	5.48 ± 0.01 a	46.52 ± 0.33 d	96.57 ± 7.99 d
WBF500	44.45 ± 3.05 c	7.79 ± 0.14 de	5.71 ± 0.29 a	30.46 ± 6.81 c	75.60 ± 5.42 b
WBF1000	44.58 ± 1.22 c	7.83 ± 0.23 e	5.70 ± 0.01 a	27.04 ± 1.70 bc	78.19 ± 10.20 bc

CF: control formulation; TBF: traditional bread flour; WBF: whole wheat bread flour. The values with the same letter in the same column do not present significant differences (*p* < 0.05).

**Table 4:** Colour parameters of cookies.

	Flours			Cookies		
	L*	a*	b*	L*	a*	b*
Control	89.94 ± 2.76 d	1.13 ± 0.17 a	10.71 ± 0.02 a	51.78 ± 6.29 d	5.68 ± 0.79 a	22.07 ± 0.73 ab
TBF1000 50%	ND	ND	ND	51.22 ± 2.50 cd	9.85 ± 0.55 b	24.79 ± 0.04 d
WBF1000 50%	ND	ND	ND	45.36 ± 0.15 abcd	9.90 ± 0.87 b	22.12 ± 0.91 abc
TBF200	84.62 ± 2.32 cd	5.91 ± 1.42 b	18.66 ± 1.04 b	50.63 ± 4.40 bcd	10.22 ± 0.08 b	23.61 ± 0.94 bcd
TBF500	81.88 ± 1.54 c	6.90 ± 1.31 b	20.08 ± 1.11 bc	44.63 ± 1.18 abc	10.44 ± 0.74 b	23.78 ± 0.04 cd
TBF1000	81.00 ± 2.22 c	7.17 ± 1.17 b	20.59 ± 1.11 c	50.42 ± 1.75 bcd	10.82 ± 0.18 bc	24.71 ± 0.08 d
WBF200	73.64 ± 2.45 b	9.56 ± 0.84 c	22.42 ± 0.34 d	44.99 ± 0.26 abc	12.01 ± 0.11 c	22.88 ± 0.33 abc
WBF500	67.98 ± 1.40 a	10.58 ± 0.75 c	23.34 ± 0.59 d	40.90 ± 2.86 a	11.06 ± 0.74 bc	21.53 ± 0.81 a
WBF1000	65.30 ± 2.78 a	10.66 ± 0.74 c	23.00 ± 0.37 d	44.50 ± 0.33 ab	11.95 ± 0.08 c	23.22 ± 0.11 abcd

CF: control formulation; TBF: traditional bread flour; WBF: whole wheat bread flour. L*: lightness; a*: green-red axis; b*: blue-yellow axis. The values with the same letter in the same column do not present significant differences (*p* < 0.05). ND: no development.

**Table 5:** Sensory acceptability of cookies.

Sample	Appearance	Odour	Taste	Texture	Overall Acceptability
Control	6.88 ± 1.62 c	6.55 ± 1.60 b	6.74 ± 1.95 c	6.40 ± 1.80 b	6.69 ± 1.49 c
TBF1000 50%	7.15 ± 1.39 cd	6.63 ± 1.43 b	7.35 ± 1.52 d	7.05 ± 1.53 c	7.28 ± 1.32 d
WBF1000 50%	7.47 ± 1,29 d	6.30 ± 1.44 b	7.10 ± 1.53 cd	7.13 ± 1.35 c	7.21 ± 1.26 d
TBF1000	6.05 ± 1.78 b	5.81 ± 1.40 a	5.27 ± 2.03 b	4.57 ± 1.83 a	5.34 ± 1.61 b
WBF1000	5.51 ± 1.76 a	5.76 ± 1.44 a	4.72 ± 2.09 a	4.14 ± 1.98 a	4.89 ± 1.61 a

CF: control formulation; TBF: traditional bread flour; WBF: whole wheat bread flour. The values with the same letter in the same column do not present significant differences (*p* < 0.05).

## Data Availability

The data that support the findings of this study are available from the corresponding author upon reasonable request.
